# A scaleable inducible knockout system for studying essential gene function in the malaria parasite

**DOI:** 10.1093/nar/gkae1274

**Published:** 2024-12-31

**Authors:** Abhinay Ramaprasad, Michael J Blackman

**Affiliations:** Malaria Biochemistry Laboratory, The Francis Crick Institute, 1 Midland Road, NW1 1AT London, UK; Malaria Biochemistry Laboratory, The Francis Crick Institute, 1 Midland Road, NW1 1AT London, UK; Faculty of Infectious and Tropical Diseases, London School of Hygiene & Tropical Medicine, Keppel Street, WC1E 7HT London, UK

## Abstract

The malaria parasite needs nearly half of its genes to propagate normally within red blood cells. Inducible ways to interfere with gene expression like the DiCre-lox system are necessary to study the function of these essential genes. However, existing DiCre-lox strategies are not well-suited to be deployed at scale to study several genes simultaneously. To overcome this, we have developed SHIFTiKO (frameshift-based trackable inducible knockout), a novel scaleable strategy that uses short, easy-to-construct, barcoded repair templates to insert *loxP* sites around short regions in target genes. Induced DiCre-mediated excision of the flanked region causes a frameshift mutation resulting in genetic ablation of gene function. Dual DNA barcodes inserted into each mutant enables verification of successful modification and induced excision at each locus and collective phenotyping of the mutants, not only across multiple replication cycles to assess growth fitness but also within a single cycle to identify specific phenotypic impairments. As a proof of concept, we have applied SHIFTiKO to screen the functions of malarial rhomboid proteases, successfully identifying their blood stage-specific essentiality. SHIFTiKO thus offers a powerful platform to conduct inducible phenotypic screens to study essential gene function at scale in the malaria parasite.

## Graphical Abstract

**Graphical Abstract:**
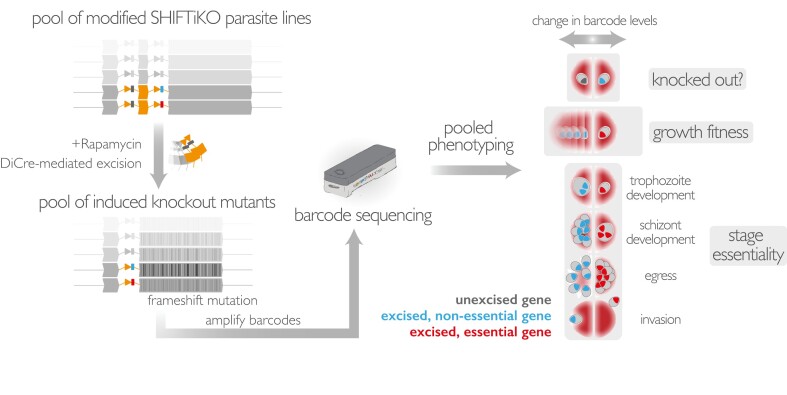

## Introduction

The ability to manipulate genes in the malaria-causing parasite, *Plasmodium*, plays a major role in efforts to study the biology of this parasite in the laboratory (1). This remains highly relevant as malaria continues to be a major global health problem causing 608 000 deaths in 2022 (2). Emerging resistance to frontline antimalarial drugs further threatens concerted efforts to reduce the malaria disease burden. Over 35% of the ∼5000 protein-coding genes in the genome of this early-diverging eukaryote lack any known functional domains (3) that might inform on their putative function. As a result, progress in understanding of *Plasmodium* biology relies upon the pace at which researchers can interrogate and discover gene function in this parasite (4).

Systematic study of gene function is challenging in *Plasmodium*. Despite work over the past decade successfully adapting CRISPR-Cas9 technology in the parasite (5,6), the lack of canonical non-homologous end-joining machinery (7) and the suboptimal efficiency of integrating exogenous DNA into the genome through homologous recombination still hinders our capability to perform gene disruption at scale. These challenges notwithstanding, immense efforts have screened gene essentiality at the genome scale using gene targeting vector libraries in the rodent malaria parasite *P. berghei* (8) and non-targeted transposon mutagenesis in the most lethal human malaria parasite *P. falciparum* (9). These screens have revealed that the parasite requires nearly half of its genes to propagate normally during the clinically relevant asexual blood stages, which involves successive cycles of intraerythocytic replication. Conditional gene manipulation is necessary to study these essential genes (10). Malaria parasites are haploid during the blood stages and so disruption of an essential gene renders the mutant non-viable for further study. To address this, several inducible strategies have been implemented in *P. falciparum* to precisely regulate gene expression by inducing changes at the DNA (11), RNA (12,13) or protein (14–16) level through the addition or removal of regulatory small molecules. Since parasite viability is not affected until the point of induction, these strategies allow an essential gene to be inactivated in a population of parasites at a desired time point and the resulting phenotype to be subsequently monitored.

One such inducible approach is the Dimerisable Cre (DiCre)-lox system in which a gene or critical sequence thereof can be inducibly deleted from the genome to disrupt its function using the site-specific recombinase Cre. Inducible control of Cre activity is brought about by constitutively expressing Cre in the parasite as two separate, enzymatically inactive polypeptides, respectively fused to rapamycin-binding FKBP12 and FKB proteins. A target region containing the gene of interest is marked for removal by flanking it with short, 34 bp *loxP* sequences that are recognizable by Cre. Addition of the drug rapamycin (RAP) dimerises the two Cre subunits to restore recombinase activity, and the active DiCre excises the *loxP*-flanked (‘floxed’) sequence. This process is highly efficient, capable of excising the target locus in close to 100% of parasites in a population upon treatment with RAP and can result in complete ablation of the gene’s expression to produce a definitive observable phenotype within the same or subsequent erythrocytic cycle (11,17). In important refinements of this technology, artificial *loxP*-containing intron modules (*loxPint*) (18) that can be used to silently introduce *loxP* sites within an open reading frame and the use of CRISPR-Cas9 to flox sequences (17) now allows the selective targeting of any region within any gene to rapidly generate inducible knockout mutants in *P. falciparum*. These combined strategies have been highly effective in studying genes with indispensable roles in parasite development (19,20), egress (21,22), invasion (23,24) and pathogenesis (25,26) during blood stages, and sensitive enough to discern even mild phenotypic defects in the case of some non-essential genes (27,28).

Whilst useful for detailed functional analysis of individual genes, the current workflow is not scaleable for functional screening of larger cohorts of genes for a number of reasons. To flox a region within a gene, it must be replaced with a ‘recodonized’ version incorporating silent base substitutions in order to retain gene function prior to excision and to protect from repeated Cas9-mediated cleavage at the target sites (17). Commercial synthesis of these gene repair constructs at scale can be expensive and requires long turnaround times depending on the length of the floxed region. Suboptimal transfection efficiency in *P. falciparum* means that successfully modified parasite clones have to be isolated and expanded to a workable culture volume before gene excision can be induced, which can take several weeks (29). Once excision of the floxed region is confirmed by PCR, phenotyping must ensue through growth assays that measure mutant growth fitness and phenotypic assays that reveal the nature of the limiting phenotypic defect using microscopy and flow cytometry. These experiments require extensive time, effort and reagents, which can be prohibitive for targeting multiple genes. Whilst the use of selection-linked integration (16), an approach that improves the rates of obtaining modified lines in *P. falciparum*, has enabled systematic generation of several inducible knockout lines (25,30), costly synthesis of recodonized regions and resource-intensive phenotyping still limits the use of this approach. Therefore, the current DiCre-lox strategy must be reimagined if we are to perform inducible gene knockouts at scale in the malaria parasite.

Here, we have developed a frameshift-based trackable inducible knockout (SHIFTiKO) system that uses short, modular repair templates to flox small regions within genes and simultaneously tag them with DNA barcodes. Induced excision introduces a frameshift mutation that renders the gene non-functional and the incorporated barcodes enable phenotyping of several mutants in a pooled manner. We demonstrate the utility of SHIFTiKO as a scaleable refinement to the DiCre-lox system by screening the functions of a family of rhomboid proteases in *P. falciparum*.

## Materials and methods

### Repair template design and plasmid construction

To create *boxit-* and *boxit+* modules, we used two heterologous *loxPint* sequences, SERA2:*loxPint* (18) and SUB2:*loxPint* (31) created previously by placing the 34 nt *loxP* sequence in endogenous introns belonging to *sera2* and *sub2* genes respectively. These introns were selected because they were short, contained only a few extended mono-nucleotide tracts and therefore could be readily synthesized artificially. The *loxP* sites were inserted in them without affecting their probable branchpoints. We introduced further sequence changes around these *loxP* sites, reasoning that the region which already includes a 34 bp *loxP* sequence would be able to sustain a few more changes without disrupting intron function. Four basepairs were modified upstream of the *loxP* site to produce the forward primer-binding site for barcode amplification (barcode.F) in *boxit-* alone, whilst a 12 bp unique barcode followed by a 19 bp reverse priming-binding site (barcode.R) were inserted after the *loxP* site in both *boxit-* and *boxit+*.

All repair templates in this study were designed as follows. Two high-quality guide RNA (gRNA) sequences in close proximity (<200 bp) to each other and upstream of a gene’s functional domain were chosen from gRNAs predicted using EuPaGDT (32). A region that spans the two Cas9 cleavage sites and of length not divisible by three was chosen as the target segment to be recodonized and floxed (FR), and the surrounding ∼500 bp regions were chosen as homology arms (RHA and LHA). For rhomboid genes, a triple hemagglutinin (3xHA) tag and an additional recodonized sequence of the region spanning the start codon to the FR (RR) was added to the template. The repair templates, LHA:*boxit-*:FR:*boxit+*:RHA (or LHA:3xHA:RR:*boxit-*:FR:*boxit+*:RHA), were fully synthesized commercially (Azenta Life Sciences) for all target genes except for *gap45*, *gdpd* and *piplc*. These genes were used as test cases to develop modular one-pot assembly protocols for building repair plasmids.

To construct repair templates for *gap45*, *gdpd* and *piplc*, a two-step overlap-extension PCR (OE-PCR) was used. To simplify the steps, the *boxit-*:FR:*boxit+* sequence was synthesized with 25 bp overhang sequences homologous to the ends of the left and right homology arms, so that the synthetic construct could be used directly without prior amplification with column-purified amplified homology arms acting as primers in the first PCR reaction. The resulting assembled amplicon was then selectively amplified in the second PCR reaction using outer primers of the homology arms and cloned into the pCR-Blunt vector using Zero Blunt PCR cloning kit (ThermoFisher Scientific). Another set of repair templates with longer homology arms were constructed with a modular one-pot In-Fusion (TakaraBio) assembly approach by taking advantage of the common *boxit* sequences. The ∼550 bp long *boxit-*:FR:*boxit+* synthesized segments were amplified using a common primer pair, boxit.F and boxit.R, and the two ∼800 bp long homology arms were amplified from parasite genomic DNA with primers carrying common 20 bp extensions (Supplementary Figure S1B and C). Column-purified amplicons were then assembled into a standard backbone vector in an In-Fusion reaction, according to kit instructions. In this study, repair templates produced by OE-PCR were ultimately used for transfections, however, we now prefer the shorter one-pot In-Fusion method for robust repair plasmid assembly.

The dual-guide targeting plasmid (pCas9-Duo) was designed based on a previously published strategy (33). The gRNA expression cassette in pDC2-Cas9-gRNA-h*dhfr* (human dihydrofolate reductase)-y*fcu* (yeast cytosine deaminase/uridyl phosphoribosyl transferase) targeting plasmid (17) was duplicated (gRNA2) but with the ends of the gRNA insertion site modified (TTGG and AAAT in gRNA2 instead of ATTG and CAAA in gRNA1 respectively). To create each targeting plasmid, two gRNA sequences were ordered as oligos with the above-specified overhangs, annealed and inserted into pCas9-Duo in a simple one-pot Golden Gate assembly. The second gRNA cassette is ideally placed near the binding site of M13 forward primer, so that successful insertion of gRNA1 and gRNA2 can be confirmed by Sanger sequencing using M13.R and M13.F_R primers respectively.

CloneAmp HiFi PCR Premix (TakaraBio) was used for all PCR reactions. gRNA sequences in the targeting plasmid were confirmed by Sanger sequencing (Azenta Life Sciences) and repair template sequences were confirmed by long-read sequencing (Plasmidsaurus). For sequences of oligonucleotides and other synthesized sequences used in this study, please refer to Supplementary Table S1.

### Parasite culture maintenance, synchronization and transfection

The DiCre-expressing *P. falciparum* B11 line (34) was maintained at 37°C in human red blood cells (RBCs) in RPMI 1640 containing Albumax II (Thermo Fisher Scientific) supplemented with 2 mM L-glutamine. Synchronization of parasite cultures were done as described previously (35) by isolating mature schizonts by centrifugation over 70% (v/v) isotonic Percoll (GE Healthcare, Life Sciences) cushions, letting them rupture and invade fresh erythrocytes for 2 h at 100 rpm, followed by removal of residual schizonts by another Percoll separation and sorbitol treatment to finally obtain a highly synchronized preparation of newly invaded ring-stage parasites.

To obtain *shiftiko* lines, transfections were performed by introducing 20 μg of targeting plasmid and 60 μg of linearised repair template (purified using PureLink™ HiPure Plasmid Midiprep Kit, Invitrogen) into ∼10^8^ Percoll-enriched schizonts by electroporation using an Amaxa 4D Nucleofector X (Lonza), using program FP158 as previously described (36). Drug selection with 2.5 nM WR99210 was applied 24 h post-transfection for 4 days with successfully transfected parasites arising at 2–3 weeks. Individual *shiftiko* lines were cryopreserved prior to pooling and starting the SHIFTiKO workflow.

### SHIFTiKO workflow

To create a pooled culture, equal starting proportions were not sought after as this would require Percoll synchronizations and haematocrit measurements for each culture. To simplify the procedure, *shiftiko* lines were treated with sorbitol to roughly synchronize them to ring or young trophozoite stages, parasitaemia was estimated using a flow cytometer (see Growth and phenotypic assays) and parasites from each line were added to a fresh culture of RBCs aiming for only roughly equal numbers. The mixed culture was continuously cultured to attain 5–10% parasitaemia. To induce DiCre-mediated excision in this pool, Percoll-synchronized early rings of about 3–5% parasitaemia were treated with RAP (10 nM overnight). Mock (DMSO) treated parasites were used as non-induced controls. To obtain mature schizonts at the end of cycle 0, ∼45 h schizonts were Percoll-isolated and allowed to mature by arresting egress using the PKG inhibitor 4-[7-[(dimethylamino)methyl]-2-(4-fluorphenyl)imidazo[1,2-α]pyridine-3-yl]pyrimidin-2 amine ( compound 2, C2, 1 μM) for 3 h. To allow these parasites to then undergo egress and invasion, egress-stalled schizonts were washed with fresh media to remove C2 and added to fresh erythrocytes (2% haematocrit) and allowed to invade for 4 h with mechanical shaking (100 rpm). Newly formed rings and the remaining non-egressed schizonts were separated using a Percoll cushion and the rings fraction was further treated with sorbitol to remove residual schizonts. At each time point specified in Figure 3B, culture volumes yielding 20–30 μl packed parasitized RBCs or 10–15 μl isolated schizonts were collected and frozen.

**Figure 1.**
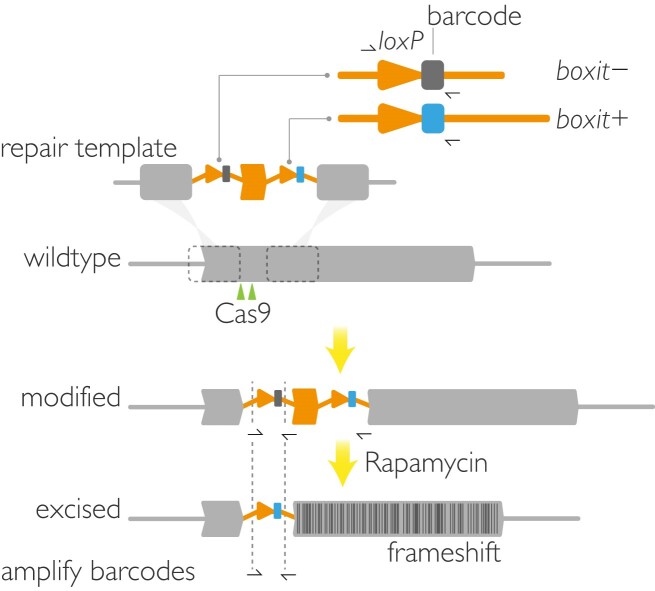
SHIFTiKO design. A short segment of length not divisible by three is replaced in the target gene with a recodonized sequence flanked by a pair of synthetic intron modules (*boxit*), each containing a *loxP* site (arrow head) and a unique 12 bp barcode. Gene modification is achieved by a Cas9-driven homology directed repair strategy in which double-stranded breaks are introduced in two gRNA target sites (shown near "Cas9") simultaneously. Treatment with rapamycin induces DiCre-mediated excision of the segment, resulting in a frameshift mutation to render the downstream segment of the gene non-functional. Binding sites for barcode-amplifying primers, barcode.F and barcode.R (half arrows) are placed in such a way that only the *boxit-* barcode is amplified from a modified *shiftiko* line and only the *boxit+* barcode is amplified from the excised mutant.

**Figure 2.**
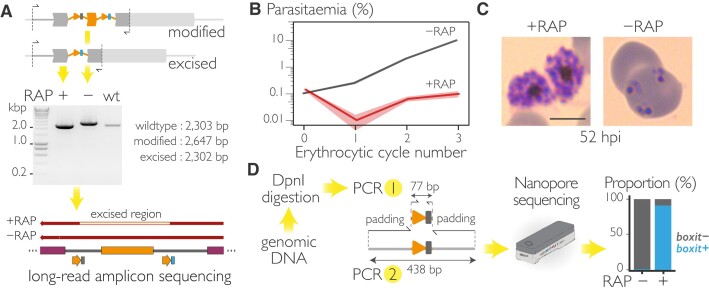
Induced disruption of SUB1 function using SHIFTiKO. (**A**) Diagnostic PCR amplification of the entire modified locus from *sub1-shiftiko* parasites at 24 h following RAP or mock treatment confirms efficient excision. Expected amplicon sizes are indicated. Long-read sequencing of the amplicons show precise excision of the floxed region containing the *boxit-* barcode whilst the *boxit+* barcode is retained in the RAP-treated parasites. (**B**) Treatment with RAP results in ablation of growth in an uncloned transfectant population of *sub1-shiftiko* parasites. Data shown are averages from three biological replicates using different blood sources (shaded ribbon, ± SEM). (**C**) Light microscopic images of Giemsa-stained *sub1-shiftiko* parasites at 52 h following RAP and mock treatment at ring stages show that RAP-treated mutants are unable to undergo egress. Scale bar, 5 μm. (**D**) To amplify barcodes from RAP- and mock- treated *sub1-shiftiko* parasites, genomic DNA extracts were first digested with DpnI to selectively remove residual repair plasmids carried over from the transfectant cultures. Nested PCR results in 438 bp long amplicons that were sequenced on a ONT MinION device. Barcode counts show increase in *boxit+* proportions in the RAP-treated population signifying efficient excision.

**Figure 3.**
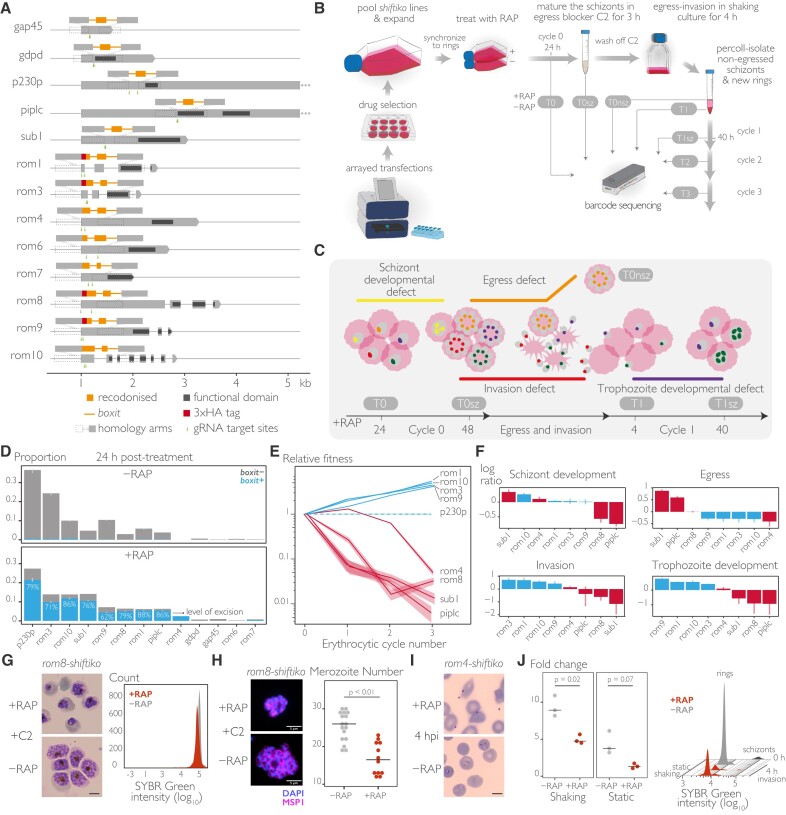
Inducible phenotypic screening of *Plasmodium* rhomboids using SHIFTiKO. (**A**) Uniform floxing strategies to create *shiftiko* lines for the 13 target genes (for gene IDs, see Supplementary Figure S2). In each case, a short region upstream of the functional domain and containing two good scoring gRNA target sites was chosen to be floxed with *boxit* modules. Simultaneous tagging of the N-terminus with a 3xHA tag was successful for four of the eight rhomboids attempted. (**B**) SHIFTiKO workflow to assess fate of several inducible mutants during blood stage progression. Transfections and subsequent drug-selection for transfected parasites are carried out in arrayed format to produce individual *shiftiko* lines, that are then pooled together, expanded and synchronized to set up a culture of tightly synchronized ring stages. Following rapamycin (RAP) treatment, samples are collected at carefully chosen time points during four successive erythrocytic cycles to capture the informative changes in barcode abundances : Mock- (−RAP) and RAP-treated (+RAP) samples from 24 h post-treatment (T0) to inform on successful integration and excision; fully mature +RAP schizonts from cycle 0 (T0sz), newly formed rings (T1) and the remaining non-egressed schizonts (T0nsz) after letting +RAP schizonts from cycle 0 undergo egress and invade fresh RBCs, and +RAP schizonts from cycle 1 (T1sz) to inform on knockout phenotypes; and samples from cycle 2 and 3 (T2 and T3) in addition to T0 and T1 to inform on growth fitness. Barcodes are amplified from genomic DNA from the collected samples and sequenced on a ONT MinION device. (**C**) Rationale behind choosing sampling time points within cycle 0 and 1 to evaluate knockout phenotypes. Mutants with a defect in schizont (yellow) or trophozoite (purple) development are expected to have fewer DNA copies and therefore lower barcode abundances in T0sz and T1sz respectively, compared to non-defective mutants (green). Barcodes of mutants with an invasion defect (red) are expected to be depleted in newly formed ring fractions (T1) whilst those of mutants with an egress defect (orange) are selectively enriched in the non-egressed schizont fraction (T0nsz). (**D**) Proportions of *boxit-* and *boxit+* barcodes in mock- (−RAP) and RAP-treated (+RAP) parasites (T0) show successful integration and excision in 9 out of 13 target genes. Data shown are averages from three replicate RAP treatments (error bars, ± SEM). (**E**) Relative growth fitness of knockout mutants (changes in *boxit+* barcode proportions from T0, normalized to the non-essential *p230p* gene) reveal essential (red) and non-essential (blue) genes (shaded ribbon, 95% confidence interval of the ratio estimated using delta method). (**F**) Within-cycle changes in barcode proportions (log_2_ ratios normalized to the non-essential *p230p* gene) reveal knockout phenotypes for the mutants (red, essential gene, blue, non-essential). Inferring the phenotypes from the first instance of aberrant change in barcode abundances during cycle progression, data reveals a schizont developmental defect (T0sz versus T0) in *rom8* and *piplc*, an egress defect (T0nsz versus T0sz) in *sub1* and an invasion defect (T1 versus T0sz) in *rom4* mutants. Data shown are averages from three replicate experiments (error bars, ± SEM). (**G**) Light microscopic images of Giemsa-stained *rom8-shiftiko* schizonts allowed to fully mature in the presence of reversible egress inhibitor C2 following RAP (+RAP) and mock (−RAP) treatment at ring stages. ROM8-null parasites exhibit defective development producing abnormal schizonts, as confirmed and quantified using flow cytometry to measure parasite DNA content. Fluorescence intensity of the SYBR Green-stained RAP-treated population (red) was detectably lower than that of the mock-treated population (grey). Scale bar, 5 μm. (**H**) MSP1 (merozoite surface protein 1) localization in ROM8-null schizonts (+RAP) show evidence of segmentation to produce individual merozoites, albeit in significantly lower numbers compared to mock-treated controls (−RAP) (crossbar represents median; *n*= 12; Welch *t*-test with Bonferroni adjusted *P*-value). Scale bar, 5 μm. (**I**) Light microscopic images of Giemsa-stained RAP- and mock-treated *rom4-shiftiko* parasites after 4 h of invasion in static cultures. ROM4-null merozoites are unable to invade RBCs and form ring stages. Scale bar, 5 μm. (**J**) Fold change in parasitaemia after 4 h invasion of mock- (−RAP) and RAP-treated (+RAP) *rom4-shiftiko* parasites under shaking and static conditions. RAP-treated cultures show no increase in parasitaemia under static conditions but show a 5-fold increase in shaking cultures, albeit still lower than mock-treated controls (crossbar represents median, three replicate RAP treatments with different blood sources; individual points represent each replicate). Fluorescence intensity of SYBR Green-stained parasite populations pre- (0 h) and post-invasion (4 h) confirm that ROM4-null mutants undergo normal egress (causing the disappearance of the schizont peak) and that the increase in parasitaemia in shaking cultures is due to formation of ring stages.

### Barcode sequencing

Genomic DNA was extracted from collected samples using DNeasy Blood and Tissue kit (Qiagen) and DpnI-digested for 1 h. To amplify the barcodes, nested PCR was run with primer pairs barcode.F-barcode.R and padding.F-padding.R using KAPA HiFi HotStart Readymix. Gel-extracted and column-purified amplicons were repurified using AMPure beads (Beckman Coulter, 1.8X sample volume). DNA libraries were prepared using Ligation Sequencing Kit (SQK-LSK109) and Native Barcoding Expansion 1-12 (EXP-NBD104) kits, pooled and sequenced either in a MinION R9 flow cell or a Flongle R9 flow cell according to manufacturers’ instructions.

### Growth and phenotypic assays

All assays on individual *shiftiko* lines were performed on uncloned populations. Growth assays were performed to assess parasite growth across 3–4 erythrocytic replication cycles. Synchronous cultures of ring-stage parasites at 0.1% parasitaemia and 2% haematocrit were maintained in triplicate in 48-well plates. Fifty microliter from each well was sampled at 0, 2, 4 and 6 days post-RAP treatment (roughly when the parasites are at 24 h into their replication cycle), fixed with 50 μl of 0.2% glutaraldehyde in phosphate buffered saline (PBS) and stored at 4°C for flow cytometry quantification. Fixed parasites were stained with SYBR Green (Thermo Fisher Scientific, 1:10 000 dilution) for 20 min at 37°C and analysed by flow cytometry on a BD FACSVerse using BD FACSuite software. For every sample, parasitaemia was estimated by recording 10 000 events and filtering with appropriate forward and side scatter parameters and gating for SYBR Green stain-positive (infected RBCs) and negative RBCs using a 527/32 detector configuration. All data were analysed using FlowJo software.

In the above experiments, samples were also collected 24 h post-RAP treatment for anaylsis by diagnostic PCR on extracted genomic DNA (DNAeasy Blood and Tissue kit, Qiagen) to confirm successful integration and excision. Amplicons from whole locus amplification were gel-extracted and sequenced (Plasmidsaurus).

To assess invasion rates in *rom4-shiftiko* parasites, highly synchronous mature schizonts were added to fresh RBCs (2% haematocrit) and allowed to invade for 4 h under either static conditions or with mechanical shaking (100 rpm) (three replicates in each condition). Cultures were sampled before and after the 4 h invasion period and fixed as before for quantification.

### Fluorescence microscopy

For immunofluorescence assays of *rom8-shiftiko* parasites, thin films of parasite cultures containing C2-arrested mature schizonts were air-dried, fixed in 4% (w/v) formaldehyde for 30 min (Agar Scientific Ltd.), permeabilized for 10 min in 0.1% (w/v) Triton X-100 and blocked overnight in 3% (w/v) bovine serum albumin in PBS. Slides were probed with anti-MSP1 human monoclonal antibody (X509; 1:1,000 dilution), followed by AlexaFluor 647-conjugated anti-human antibodies (Invitrogen, 1:1000). Slides were then stained with 1 μg/ml DAPI and mounted in Citifluor (Citifluor Ltd., Canterbury, U.K.).

Z-stacks (125-nm Z-step) were acquired on an VT-iSIM superresolution imaging system (Visitech International), using an Olympus IX83 microscope, 150×/1.45 Apochromat objective (UAPON150XOTIRF), ASI motorized stage with piezo Z, and Prime BSI Express scientific complementary metal oxide semiconductor camera (Teledyne Photometrics). The microscope was controlled with Micro-Manager v2.0 gamma software.

### Data analysis and visualization

Target gene sequences and domain information were downloaded from PlasmoDB database (37) and floxing strategies and plasmids designed in SnapGene software. To visualize the floxing strategies (Figure 3A), all feature coordinates were plotted using gggenes CRAN package in R.

Barcode-sequencing (bar-seq), basecalling and demultiplexing from raw nanopore output (.fast5 or .pod5 files) was done using Guppy v3.2.2 or Dorado v0.5.0 and the reads mapped onto the 438 bp long amplicon reference sequence using minimap2 v2.2 (-ax map-ont). Around 1000–35 000 quality-filtered reads were obtained for each sample, of which 84–89% were successfully mapped onto the reference. Exact matches to barcode sequences were counted from the mapped reads.

All statistical analysis and data visualization was performed in R v4.2.2 (38). Relative growth fitness was calculated by estimating the ratios or change in barcode proportions between Tn and T0 and normalized to changes in barcode proportions of the non-essential control *p230p*. 95% confidence intervals of these ratios were derived using the delta method of estimating variance. Welch *t*-test was used to compare group means and where necessary Bonferroni adjustment for multiple comparisons was applied to the p-value of statistical significance.

## Results

### SHIFTiKO design

First, we sought a floxing strategy that can be uniformly applied across genes irrespective of their size, thereby removing the need to synthesize long repair templates. We chose a recently adopted (28,39,40) frameshift-based gene disruption approach where a short segment within the target gene open reading frame, upstream of any putative functional domains, and of a length not divisible by three is floxed by inserting two closely-opposed *loxP*-containing intron (*loxPint*) modules. DiCre-mediated excision of the floxed region is predicted to result in a translational frameshift and the introduction of multiple downstream premature stop codons, thus truncating the gene and rendering it non-functional (Figure 1). Next, we aimed to tag the modified gene with a DNA barcode so that abundance of each parasite mutant can be quantified at the molecular level, thereby enabling simultaneous monitoring of the fitness of several mutants within uncloned populations. For this, a unique 12 base pair (bp) barcode was inserted next to the *loxP* site in two heterologous *loxPint* sequences [SERA2:*loxPint* (18) and SUB2:*loxPint* (31)] to create a pair of barcoded *loxP*-containing introns (or *boxit*; Figure 1). Specific primer-binding sites for barcode amplification were also added in such a way that a 77 bp long amplicon will contain the barcode from the first flanking *boxit* (*boxit*-) pre-excision and the barcode from the second flanking *boxit* (*boxit+*) post-excision of the floxed region. Finally, a new dual-guide targeting plasmid (pCas9-Duo) was generated that expresses two distinct gRNAs in order to enhance the chances of a successful Cas9-mediated modification at the target region (Supplementary Figure S1A). Presently, this failsafe is achieved by multiple transfections, each using different single-guide targeting plasmids.

### SHIFTiKO creates barcode-trackable inducible mutants

To test our strategy, we targeted the *sub1* gene (PF3D7_0507500), which encodes a serine protease essential for egress of blood stage parasites (21). To do this, a 199 bp segment upstream of the subtilisin-like catalytic domain was floxed with *boxit* modules. Treatment with RAP resulted in efficient DiCre-mediated excision of the floxed sequence (Figure 2A) and the loss of SUB1 expression, which as expected proved lethal to the parasite (Figure 2B) due to a severe egress defect (Figure 2C). Interestingly, diagnostic PCR of the entire target locus, whilst amplifying the modified locus (2647 bp in -RAP, Figure 2A), did not produce a smaller amplicon (2303 bp) from the unmodified locus which is generally expected when sampling transfectant populations that consist of both modified and wildtype parasites. This suggests a high rate of gene modification, possibly a result of expressing two gRNAs simultaneously and using short repair templates in SHIFTiKO. The transfectant parasites phenocopied clonal SUB1 iKO lines (21) upon treatment with RAP indicating that SHIFTiKO can produce near-homogenous populations of inducible mutants suitable for direct phenotyping.

Next, we wanted to test whether the incorporated barcodes can be used to quantify the mutants, which in this case means distinguishing between excised and unexcised parasites using the *boxit+* and *boxit-* barcodes respectively. Barcodes were amplified from genomic DNA extracted from RAP- and mock-treated *sub1-shiftiko* parasites at 24 h post-treatment using a generic pair of primers (barcode.F and barcode.R), yielding a 77 bp product. To explore the use of nanopore sequencing (Oxford Nanopore Technologies or ONT) to analyse these barcodes, we further performed nested PCR to add a random padding sequence on either side of the amplicon, thereby increasing its size to 438 bp to meet ONT’s minimum fragment size specifications. This step can now possibly be skipped with the new ‘Short Fragment Mode’ configuration (released during the course of this study: https://nanoporetech.com/news/news-oxford-nanopore-releases-short-fragment-mode-new-tool-real-time-sequencing-short) that allows sequencing of fragments as short as 20 bp. bar-seq clearly showed a rise in *boxit+* barcodes in the RAP-treated population, measuring an excision rate of 91% at the time point of sampling (Figure 2D). Taken together, these results show that *boxit* modules can be efficiently inserted into a gene without affecting its function to prime it for an effective knockout by induced frameshift mutation and the proportion of mutants in the resulting parasite population can be quantified at any point by bar-seq.

### SHIFTiKO enables inducible phenotypic screens in *P. falciparum*

Encouraged by the above results, we wanted to explore whether several *shiftiko* mutants can be phenotyped simultaneously by tracking their barcodes in a mixed population. As the test gene cohort for this work, we chose to target a family of *Plasmodium* genes encoding rhomboids, intramembrane proteases that cleave membrane-bound substrates and that are known to play important roles in protozoan parasites (41,42). Whilst previous work has suggested that four to five of the eight *Plasmodium* rhomboids are likely essential (8,9,43) (Supplementary Figure S2), a systematic study to identify the precise biological functions of these putative enzymes is lacking. To ensure that the approach used was capable of discriminating a range of growth phenotypes, we included as controls in our analysis additional well-characterized genes displaying four essential knockout phenotypes previously examined with the DiCre system, namely a limiting defect during trophozoite development [*gdpd* (20); PF3D7_1406300], schizont development [*pi-plc* (19); PF3D7_1013500], egress [*sub1* (21); PF3D7_0507500] and invasion [*gap45* (34); PF3D7_1222700], and a non-essential phenotype [*p230p* (44); PF3D7_0208900]. Uniquely barcoded *boxit* modules were inserted into the 13 target genes to flox a region of length varying from 80 to 227 bp (Figure 3A). In the case of the rhomboid genes, we also explored the possibility of simultaneously tagging the N-terminus of each protein with a triple-hemagluttinin (3xHA) epitope to aid downstream detection of the protein. To do this, we modified our strategy slightly to include a 3xHA sequence and a second recodonized sequence to replace the region extending from the start codon to the segment to be floxed (Figure 3A). After transfecting parasites with *shiftiko* plasmids and selecting them using the antifolate drug WR99210 (WR), we observed WR-resistant parasites in 9 out of the 13 cultures within 2–3 weeks. For the transfections that did not generate drug-resistant parasites, we hypothesized that tagging the N-terminus might be deleterious in the case of these genes (*rom4*, *rom6*, *rom7* and *rom10*) and subsequently were able to acquire drug-resistant parasites using repair constructs that did not incorporate the 3xHA tag.

Ring stage parasites from each culture were combined to create a unified pool that could be continuously cultured, stage synchronized and RAP-induced to produce a mixed mutant population (Figure 3B). By analysing the barcodes from this pool at specific timepoints, we could collectively track the presence and fate of each barcode-tagged mutant following induced mutagenesis. Initially, barcodes identified in the mock- and RAP-treated pool 24 h post-treatment (T0) verified successful integration and later RAP-induced excision at all targets, except for *gdpd*, *gap45*, *rom6* and *rom7* (Figure 3D). These results were validated by diagnostic PCR performed on the individual uncloned *shiftiko* lines (Supplementary Figure S3) with unsuccessful (*gap45* and *rom6*) or low level (*rom7*) of integration or inexplicable deletions within *boxit+* in the case of *gdpd* as causes for failure. In the case of *rom4*, whilst its *boxit-* barcode could not be detected, we observed appearance of its *boxit+* barcode upon treatment with RAP. Overall, a 60–90% level of excision was observed at the target genes in the pool after 24 h of treatment with RAP (Figure 3D) that then reached 97–100% by the end of cycle 0 (Supplementary Figure S4). Subsequently, to profile the replication fitness of the successfully generated mutants, we tracked the changes in barcode abundances across different replication cycles (T0, T1, T2 and T3, corresponding to cycles 0, 1, 2 and 3 post-treatment, respectively). It was anticipated that barcodes linked to essential genes would diminish or disappear entirely due to a proliferation defect in the respective knockout mutants. This expectation was confirmed as barcodes associated with essential control genes *sub1* and *piplc* were significantly reduced whilst barcode levels were sustained for the non-essential gene control *p230p* (Figure 3E). Our analysis further identified *rom4* and *rom8* as essential rhomboids, exhibiting a similar diminishing trend in their barcodes. The outcomes of the growth fitness screen corresponded well with the growth profiles recorded for individual uncloned *shiftiko* lines upon treatment with RAP (Supplementary Figure S5), thus validating the approach. Moreover, in the case of *piplc*, whilst the presence of unmodified parasites in the RAP-treated uncloned population (evident from wildtype locus amplified by diagnostic PCR, Supplementary Figure S3) caused an uncharacteristic increase in parasitaemia in the cell-based growth assay, the barcode-based growth profile using SHIFTiKO was able to precisely discern the severe growth defect in *piplc* mutants from the confounding wildtype background.

Next, within-cycle changes in barcode abundance caused naturally by DNA replication during schizogony were exploited to evaluate the effects of gene disruption on parasite development (Figure 3B and C). We predicted that a drop in barcode abundance between ∼24 h trophozoites (T0) and mature ∼48 h schizonts (T0sz) during cycle 0 would indicate a schizont developmental defect, whilst a similar decline between ∼4 h rings (T1) and ∼40 h schizonts (T1sz) during cycle 1 would indicate a trophozoite developmental defect. This prediction was supported by the case of *piplc*, a gene previously shown to be essential for schizont development, showing a ∼1.7 fold reduction in its barcode levels at schizont stages (Figure 3F, schizont development). The essential rhomboid, *rom8* also showed a similar reduction, indicating a defect in schizont development. This was further validated by detailed phenotyping of ROM8-null mutants which showed a defect in late schizont development that results in abnormal schizont forms with a lower number of merozoites compared to mock-treated controls (Figure 3G and H). Finally, we sampled barcodes after a 4 h invasion window at the end of cycle 0 to assess invasion and egress processes. Barcodes that were enriched in nonegressed schizonts isolated following the expected period of invasion (T0nsz versus T0sz) would point to an egress defect, whilst those barcodes *exclusively* depleted in the newly formed rings (T1 versus T0sz) would indicate an invasion defect. The trend observed with barcodes from *sub1* (enriched almost 2-fold in T0nsz and severely depleted in T1), a gene absolutely essential for egress, confirmed our hypothesis (Figure 3F, egress and invasion). The trends also showed that gains made by *rom4* barcodes in the ring fractions were low given the rate of their egress, suggesting an invasion phenotype for the *rom4* mutant. As confirmation, detailed phenotyping of ROM4-null parasites revealed a profound defect in their ability to invade RBCs (Figure 3I), that could be overcome to a certain extent by mechanical shaking during invasion (Figure 3J). Incidentally, this also explains the distinct growth fitness profile for *rom4*, with the slight gain in fitness in cycle 1 (T1) evidently a result of invasion being performed under shaking conditions and the downward trend later on due to subsequent invasions occurring in static cultures. Moreover, the approach was equally capable of analysing growth fitness for both overrepresented (*p230p* and *sub1*, for example) and underrepresented (*piplc*, *rom4* and *rom8*) mutant populations in the pool. In conclusion, these data show that both growth fitness and the knockout phenotype can be evaluated with great precision in mutants of multiple genes in a single experiment using SHIFTiKO, thus paving for the first time the way for inducible phenotypic screens in *P. falciparum*.

## Discussion

Conditional mutagenesis remains the only genetic strategy for the functional analysis of essential asexual blood stage genes in malaria parasites. DiCre-mediated gene disruption, which entirely eliminates protein expression, provides a more reliable phenotype than inducible knockdown methods that only reduce protein levels, especially for proteins with enzymatic activity (10). However, resource-intensive workflows specifically designed for deploying DiCre have become the primary bottleneck that restricts our capacity to study multiple genes concurrently. With SHIFTiKO, we have now greatly enhanced the scaleability of this powerful tool by dispensing of the need for synthesizing long artificial genes, limiting dilution cloning steps and repetitive phenotypic assays. We have shown here through extensive validation that SHIFTiKO can reliably identify successful floxing, successful excision and subsequent growth fitness and specific knockout phenotype for several genes within a single experiment that is reproducible across independent RAP treatments and pooled cultures (results from another independently run experiment can be found in Supplementary Figure S6). This completely replaces several time-consuming steps in the traditional DiCre workflow such as diagnostic PCR for confirming integration and excision, and laborious cell-based growth and phenotypic assays.

In its current form, SHIFTiKO can be used to deploy DiCre at scale to conduct targeted inducible screens of moderately sized groups of essential genes, such as a protein family or enzymes in a metabolic pathway. Importantly, it is well-suited for targeting very large genes and proteins without identified functional domains, as the induced frameshift strategy operates independently of such information. Pooled phenotyping significantly reduces the time needed to determine at which stage in the parasite’s asexual replication cycle a target gene is essential. This facilitates simultaneous screening of gene cohorts with time-specific roles such as transcription factors. It also allows rapid identification of genes of interest, allowing resources to be focused on efforts to characterize them in detail whilst minimizing time spent on gene targets that turn out to be dispensable (19). For example, our screen revealed that *rom1*, *rom3* and *rom9* — genes with poor fitness scores in the transposon mutagenesis screen (9)— are, in fact, dispensable. Further detailed functional analysis can be performed by reverting back to the original *shiftiko* line but would ideally require the protein to be tagged to provide insights into expression profiles, subcellular localization and biochemical analysis (e.g. by pull-down). Whilst our work has shown that concurrent N-terminal tagging is feasible when generating *shiftiko* lines, it is more practical to design simpler repair templates initially then introduce the tag later in a subsequent gene-editing step. This is facilitated by the presence of the negative selectable marker *yFCU* in the pCas9-Duo plasmid, which is episomally carried by all *shiftiko* lines.

The ability to maintain several *shiftiko* lines simultaneously as a single pooled culture owing to the conditional nature of gene disruption offers considerable robustness and convenience. During the course of this study, we have routinely cultured these pools for many months, subjected them to Percoll-based synchronization, cryopreserved and revived them, and added additional *shiftiko* lines to make newer versions of the pool without compromising usability. The barcode sequencing protocol has been specifically tailored for use with the nanopore platform as a swift, flexible and economical approach that can be readily implemented in any laboratory setting without relying on external sequencing facilities. In our experiments, barcode amplification and library preparation could be completed within a few hours and sequenced within just 6–8 h using a MinION Flow Cell or the more cost-effective Flongle. Both the duration of the run and the choice of flow cell are adjustable based on the required number of reads, which varies depending on the complexity of the mutant pool.

The method however still suffers from certain limitations. We failed to modify four of our target genes, including genes (*gap45* and *gdpd*) that have been successfully disrupted out in the past using traditional DiCre methodology. This suggests some genes (or regions within them) may be refractory to introduction of *boxit* modules in tandem. Efforts are underway to flox a different target region in these genes. Designing the floxing strategy, synthesizing the ∼550 bp repair template and assembling both targeting and repair plasmids for each gene can still be time-intensive, thereby limiting the number of *shiftiko* lines we could produce at a time. We have partially addressed this by optimizing one-pot assemblies to produce the plasmids in a rapid and modular fashion (Supplementary Figure S1B and C) that would also facilitate automated floxing design using in silico tools such as GeneTargeter (45). We have found these protocols to robustly produce plasmid constructs for several genes in our ongoing efforts to conduct more SHIFTiKO screens. Experimenting with shorter *boxit* modules and floxed regions as well as moving to a single-plasmid gene-editing strategy (46) can further simplify this process in the future and improve SHIFTiKO’s scaleability.

The creation of trackable inducible mutant lines for the first time in *P. falciparum* opens up exciting possibilities to scale up gene function discovery in this parasite. The mutant parasite pool could be exposed to various selective pressures such as drug or substrate challenges to rapidly identify genes, disruption of which confers a selective advantage under such conditions. Additionally, technologies such as flow sorting (47) and single-cell RNA sequencing could be employed to study the cell cycle progression and transcriptional states, respectively, of these mutants in a powerful way. SHIFTiKO could also be potentially useful to conduct inducible screens in other *P. falciparum* life stages (48), rodent malaria models (49) and protozoan parasites (50) with an established DiCre system.

## Supplementary Material

gkae1274_Supplemental_Files

## Data Availability

All associated data are available as follows- analysis code and raw data at doi:10.5281/zenodo.13839816, plasmid maps and sequences at doi:10.5281/zenodo.10547131 and raw read files at doi:10.5281/zenodo.13839539.

## Notes

Present address: Abhinay Ramaprasad, Centre for Parasitology, University of Glasgow, School of Infection and Immunity, Sir Graeme Davies Building, 120 University Place, Glasgow, G12 8TA, UK.
