# Supplementary material for: A scaleable inducible knockout system for studying essential gene function in the malaria parasite
Source: Nucleic Acids Res. 2024 Dec 31;53(4):gkae1274. doi: 10.1093/nar/gkae1274 (PMC11879119; doi:10.1093/nar/gkae1274)
Supplement: gkae1274_Supplemental_Files [file gkae1274_supplemental_files.zip › Suppl_Figs_R2.pdf]

Supplementary Figures

References

51. Subudhi,A.K., O'Donnell,A.J., Ramaprasad,A., Abkallo,H.M., Kaushik,A., Ansari,H.R., Abdel-Haleem,A.M., Ben Rached,F., Kaneko,O., Culleton,R., et al. (2020) Malaria parasites regulate intra-erythrocytic development duration via serpentine receptor 10 to coordinate with host rhythms. Nat. Commun., 11, 2763.

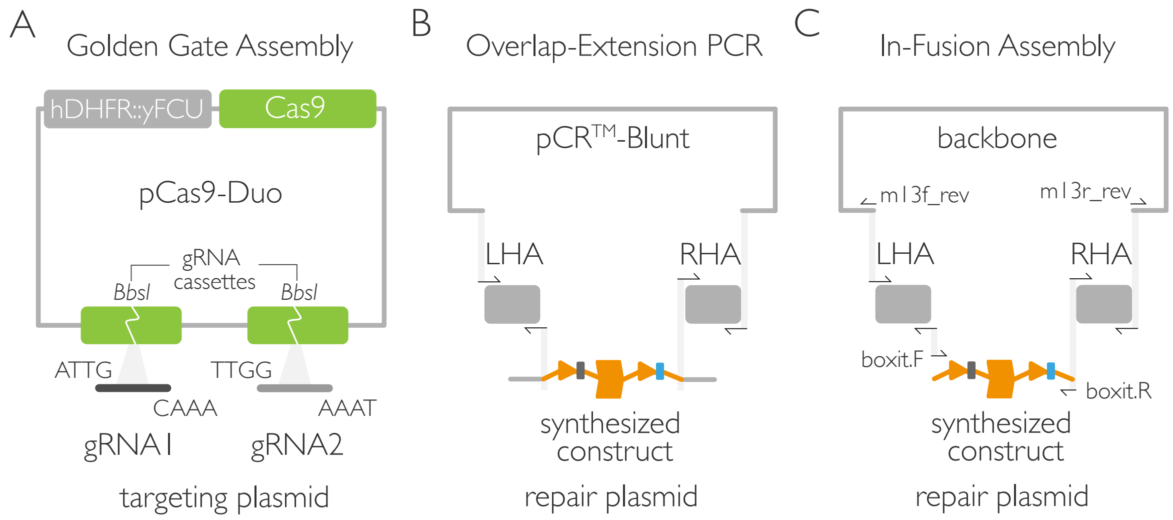

Fig. S1: **Modular assembly of SHIFTiKO plasmids.** (A) Construction of the targeting plasmid in a single-step one-pot Golden Gate assembly by inserting two annealed gRNA oligo pairs with different overhangs into the dual-guide targeting plasmid (pCas9-Duo). (B) Construction of the repair plasmid by blunt-ended cloning of the amplicon, *boxit*-:FR:*boxit+*, assembled by two-step overlap-extension PCR. (C) Construction of the repair plasmid by three-insert fragment In-Fusion cloning with uniform junction regions achieving modularity.

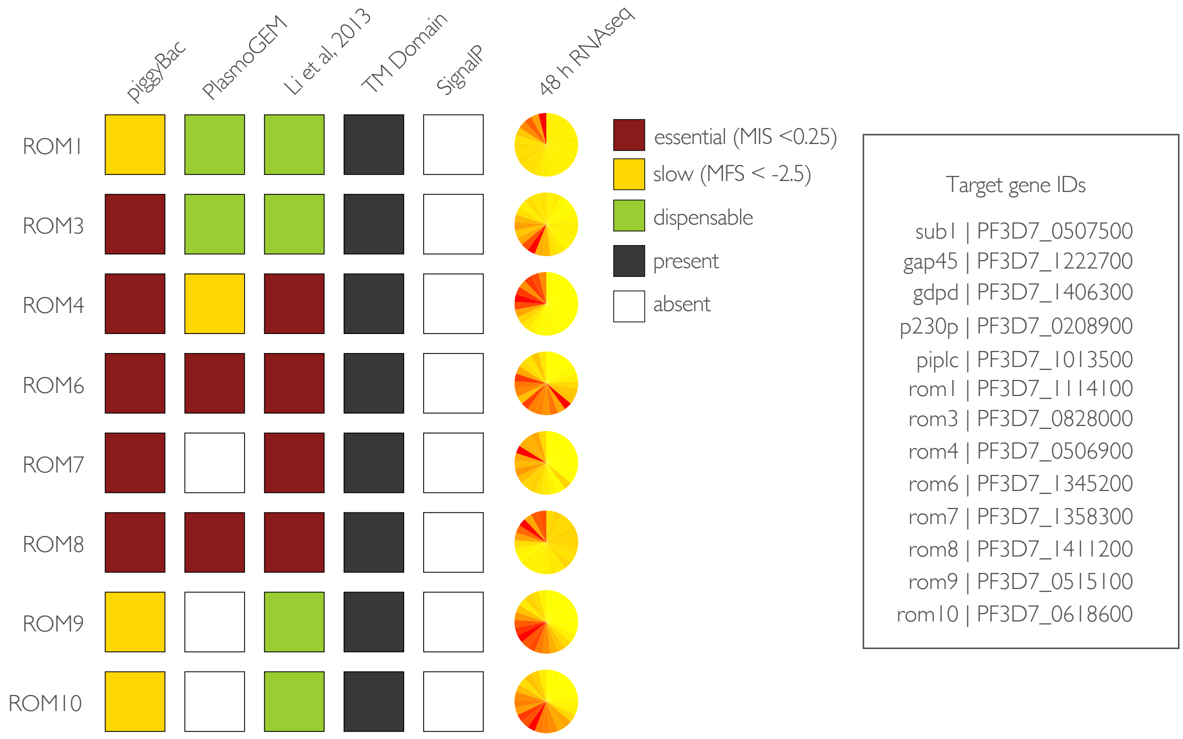

Fig. S2: **Essentiality of rhomboid proteases in *Plasmodium*** Summary of essentiality information on the eight *Plasmodium* rhomboid proteases from three studies - piggyBac [9] and PlasmoGEM [8] screens and a systematic knockout study [43]. Presence of transmembrane domains and signal peptide, and 48 h expression profile (peak expression as red in the gradient) from bulk RNAseq [51] are also shown.

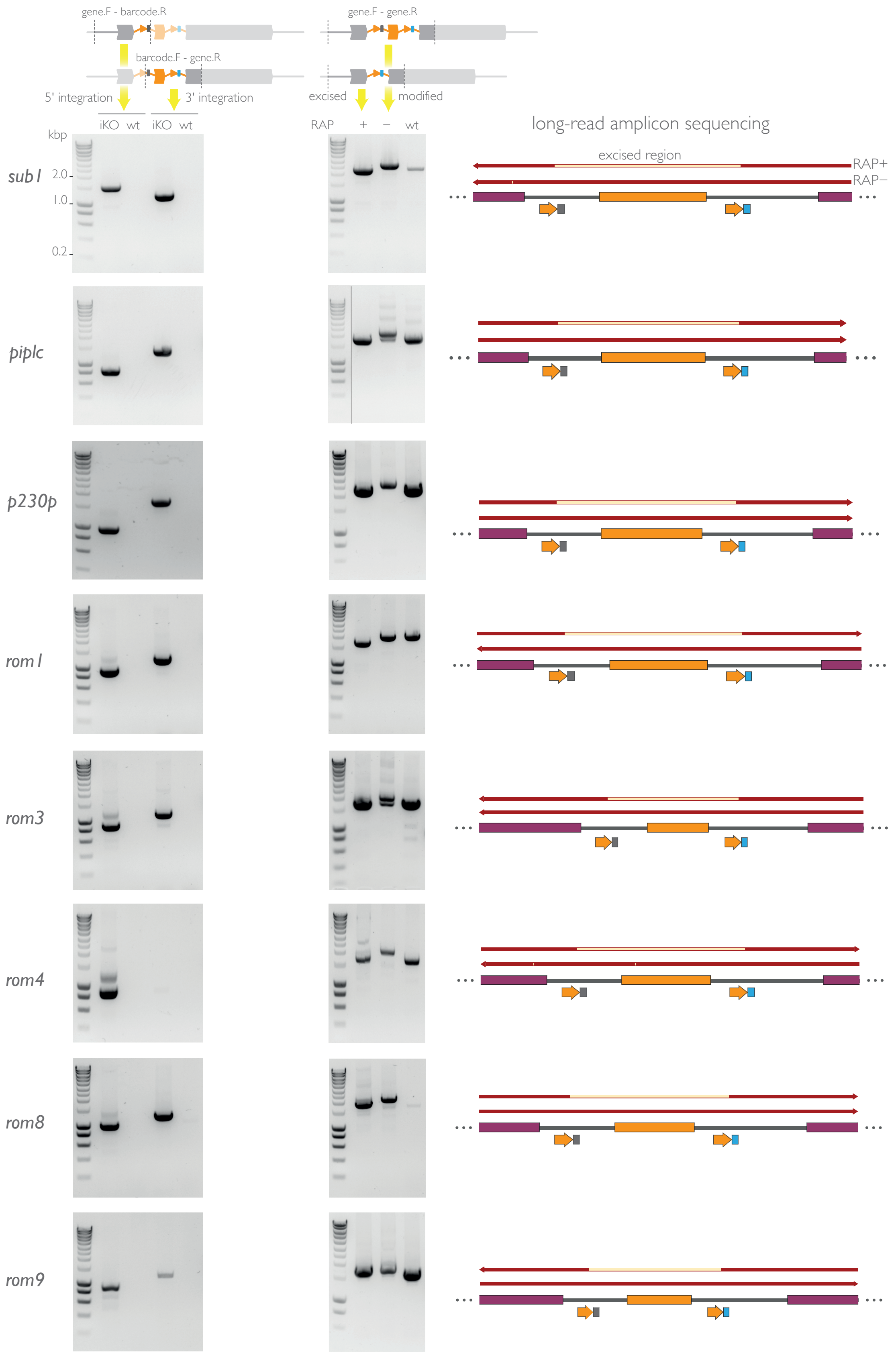
gene.F - barcode.R
barcode.F - gene.R
5' integration
3' integration
gene.F - gene.R
excised
modified
iKO wt iKO wt
RAP + − wt
kbp
2.0
1.0
0.2
long-read amplicon sequencing
excised region
RAP+
RAP−
sub1
piplc
p230p
rom1
rom3
rom4
rom8
rom9

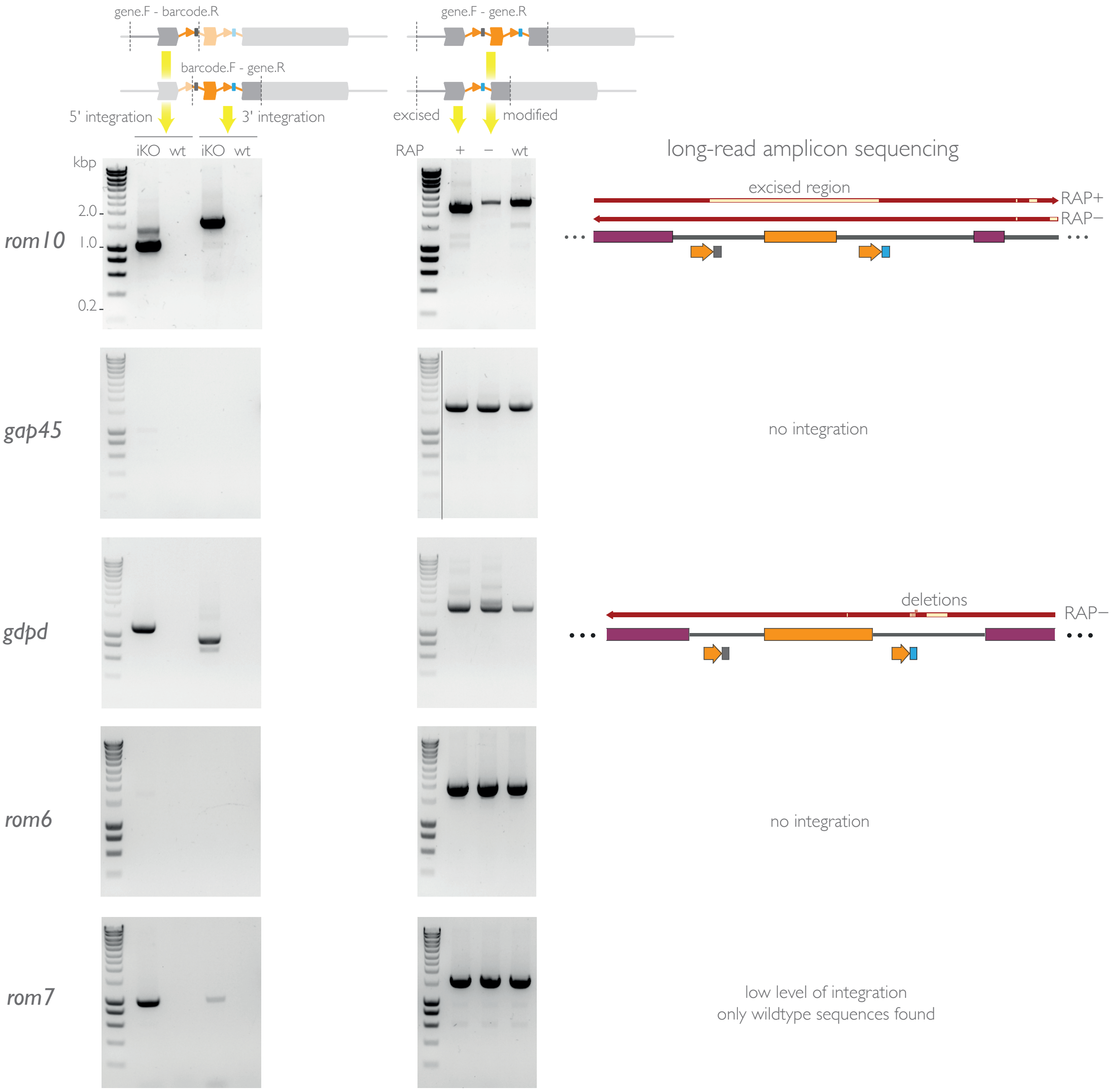

Fig. S3: **Diagnostic PCR to determine successful integration and excision in individual uncloned *shiftiko* lines.** The gel on the left shows diagnostic PCR amplification to confirm integration of repair sequence at each gene locus. Gene-specific outer primers (gene.F and gene.R) and common inner primers (barcode.F and barcode.R) were used to check for 5' and 3' integration. The gel on the right shows diagnostic PCR amplification of the entire modified locus using gene.F and gene.R primers at 24 h following RAP or mock treatment to confirm efficient excision. Further confirmation of excision was obtained by long-read sequencing of the amplicons.

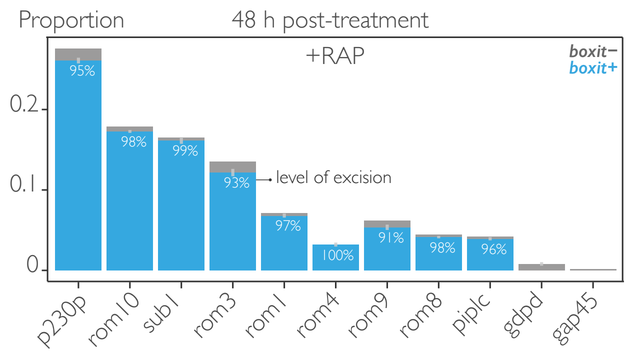

Fig. S4: **Proportions of *boxit-* and *boxit+* barcodes in mock- (-RAP) and RAP-treated (+RAP) parasites at 48 h post-treatment (T0sz).** The data shows higher levels of excision acheived after 48 h compared to 24 h (T0; Figure 3D). Data shown are averages from three replicate RAP treatments (error bars, ± SEM).

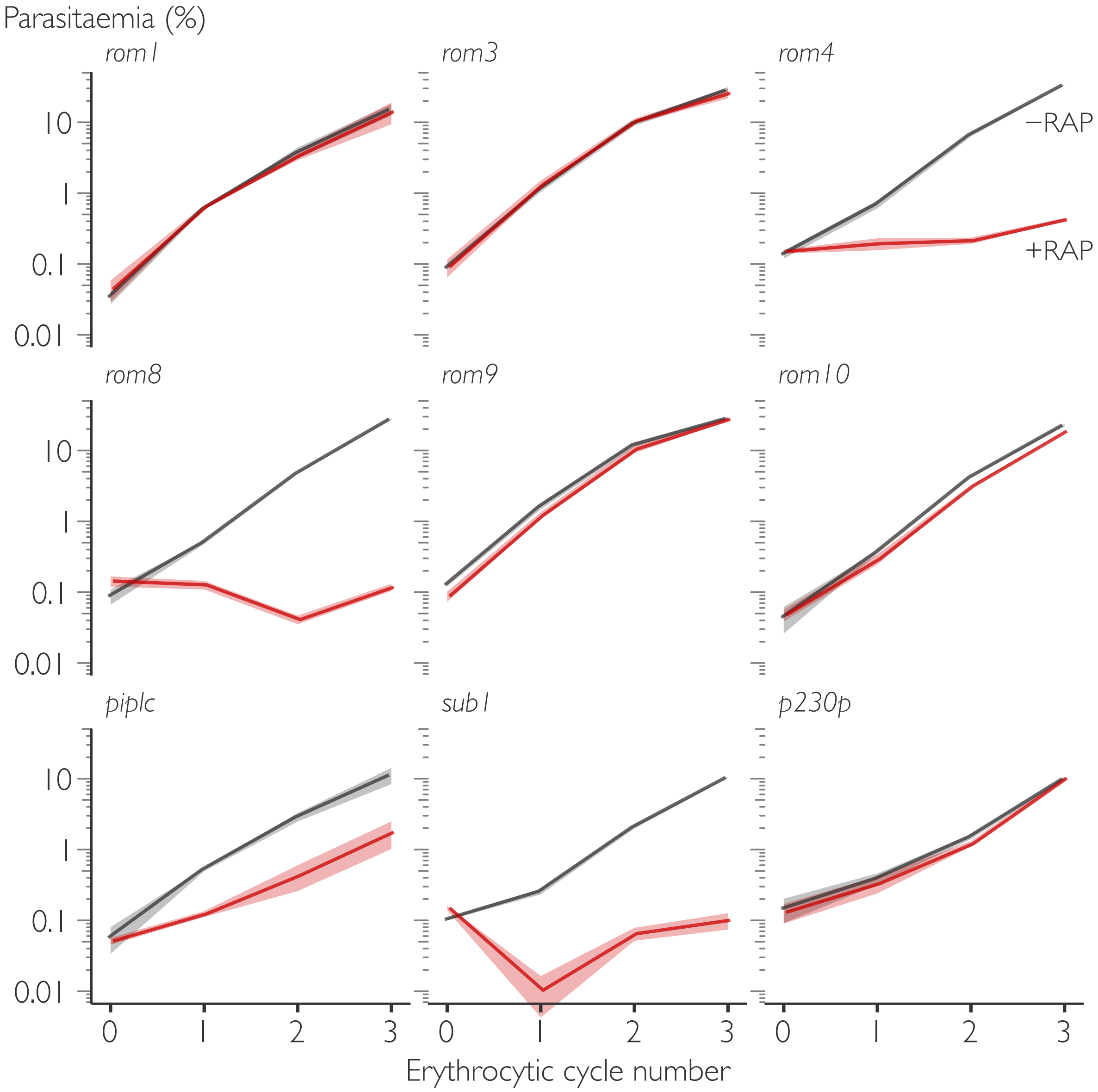

Fig. S5: **Cell-based growth profiles of mock- (-RAP) and RAP-treated (+RAP) parasites in individual uncloned *shiftiko* lines.** Data shown are averages from three biological replicates using different blood sources (shaded ribbon, ± SEM).

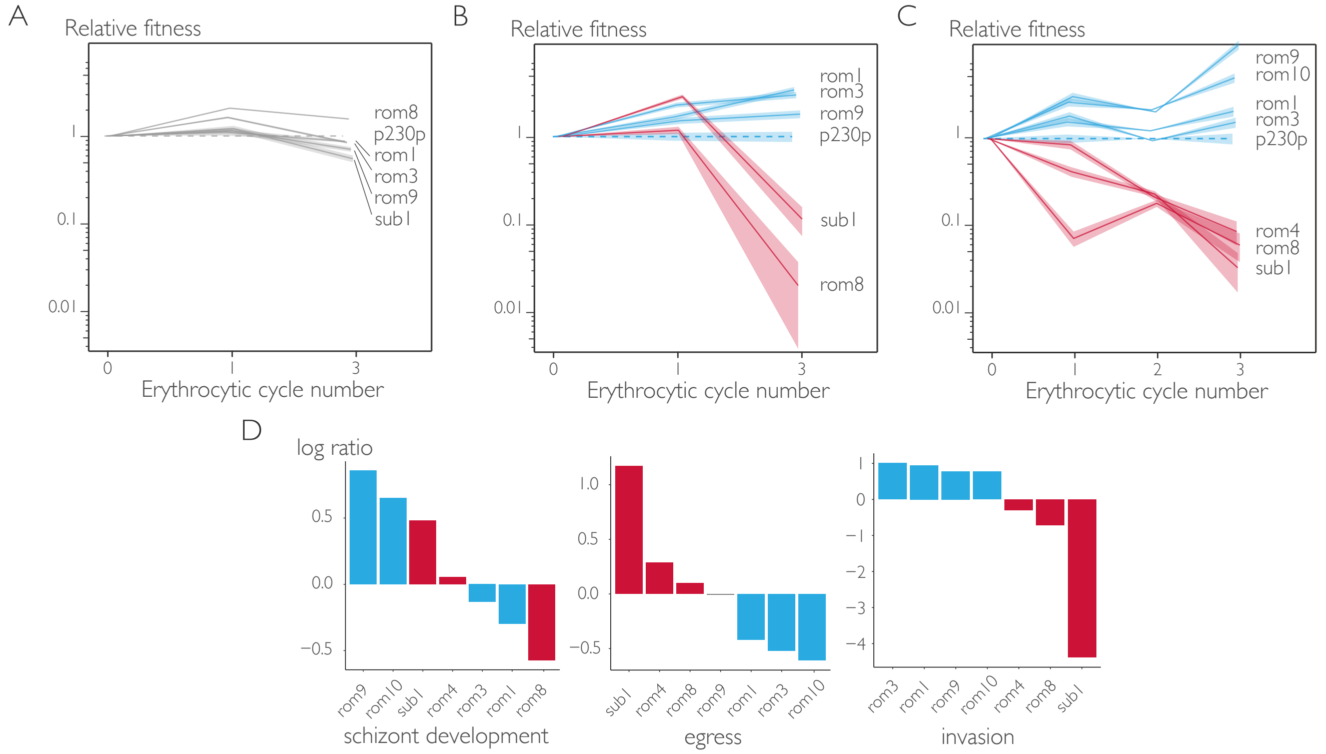

Fig. S6: **Results from inducible phenotypic screens performed on two independently constituted pools with a subset of the target genes.** (A) Relative growth fitness of untreated modified parasites in a pool with 6 *shiftiko* lines (changes in *boxit-* barcode proportions from T0, normalised to the non-essential *p230p* gene) show the parasites grow normally in the absence of induction with rapamycin. (shaded ribbon, 95% confidence interval of the ratio estimated using delta method) (B) Relative growth fitness of mutant parasites in the same pool after treatment with rapamycin (changes in *boxit+* barcode proportions from T0, normalised to the non-essential *p230p* gene) reveal essential (red) and non-essential (blue) genes. (C) Relative growth fitness of mutant parasites in another pool with 8 *shiftiko* lines after treatment with rapamycin. (D) Within-cycle changes in barcode proportions ($\log_2$ ratios normalised to the non-essential *p230p* gene) reveal knockout phenotypes for the mutants (red, essential gene, blue, non-essential). Data shown are averages from three replicate experiments (error bars, $\pm$ SEM).
